# Anti-inflammatory, analgesic and acute toxicity effects of fermented soybean

**DOI:** 10.1186/s12906-019-2791-2

**Published:** 2019-12-19

**Authors:** Hamidah Mohd Yusof, Norlaily Mohd Ali, Swee Keong Yeap, Wan Yong Ho, Boon Kee Beh, Soo Peng Koh, Kamariah Long, Noorjahan Banu Alitheen

**Affiliations:** 1 Department of Cell and Molecular Biology, Faculty of Biotechnology and Biomolecular Science, Universiti Putra Malaysia, 43400 Serdang, Selangor Malaysia; 2 China-ASEAN College of Marine Sciences, Xiamen University Malaysia, Jalan Sunsuria, Bandar Sunsuria, 43900 Sepang, Selangor Malaysia; 3 The University of Nottingham Malaysia Campus, Jalan Broga, 43500 Semenyih, Selangor Malaysia; 4 Institute of Bioscience, Universiti Putra Malaysia, Serdang, Selangor Malaysia; 5 Biotechnology Research Centre, Malaysian Agricultural Research and Development Institute (MARDI), 43400 Serdang, Selangor Malaysia

**Keywords:** Fermented, Soybean, Anti-inflammation

## Abstract

**Background:**

Tempeh is a widely known fermented soybean that contains elevated level of bioactive contents. Our previous study has shown that anaerobic fermented Nutrient Enriched Soybean Tempeh (NESTE) with increase amino acid and antioxidant levels possessed better hepatoprotective effect than raw soybean.

**Methods:**

In this study, the anti-inflammatory effect of the NESTE aqueous extract and raw soybean aqueous extract (SBE) were evaluated by quantifying the inhibition of IL-1β, TNF-α and nitric oxide (NO) secretion in LPS treated RAW 264.7 cell in vitro. On the other hand, in vivo oral acute toxicity effect of the extract was tested on mice at the dose of 5000 mg/kg body weight. In vivo oral analgesic effect of both aqueous extracts at 200 and 1000 mg/kg body weight was evaluated by the hot plate test.

**Results:**

In the in vitro anti-inflammatory study, 5 mg/mL NESTE was able to inhibit 25.50 ± 2.20%, 35.88 ± 3.20% and 28.50 ± 3.50% of NO, IL-1β and TNF-α production in LPS treated RAW 264.7 cells without inducing cytotoxic effect on the cells. However, this effect was lower than 4 μg/mL of curcumin, which inhibited NO, IL-1β and TNF-α production by 89.50 ± 5.00%, 78.80 ± 6.20% and 87.30 ± 4.00%, respectively. In addition, 1.5 to 2.5-fold increase of latency period up to 120 min for mice in the hot plate test was achieved by 1000 mg/kg NESTE. The analgesic effect of NESTE was better than 400 mg/kg of acetyl salicylic acid, which only increased ~ 1.7-fold of latency period up to 90 min. Moreover, NESTE did not show acute toxicity (no LD_50_) up to 5000 mg/kg body weight.

**Conclusion:**

NESTE is a nutritious food ingredient with potential anti-inflammatory and analgesic effects.

## Background

Inflammation is one of the naturally occurring mechanisms in the body that involves in protection against tissue injury due to physical trauma, noxious chemicals and microbial agents [1]. This response plays a vital role in the process of eliminating the invading organisms, removing the irritants and generating chemical mediators for repair of injured tissue and migrating cells [1, 2]. Macrophage is one of the immune cells that generate the chemical mediators such as reactive oxygen species (ROS) and reactive nitrogen species (RNS) when the inflammatory signalling response is activated by immune-stimulants, including bacterial endotoxin lipopolysaccharide (LPS) and interferon-gamma (IFN-γ) [3]. Nevertheless, over-expression of these mediators can subsequently induce inflammatory diseases such as rheumatoid arthritis, atherosclerosis, chronic hepatitis and pulmonary fibrosis [4, 5]. Prevention of the development of chronic inflammatory diseases could be inhibited via suppression of the over-expressed mediators [5]. Common treatment used to treat inflammatory diseases is via non-steroidal anti-inflammatory drugs (NSAIDs), which has reported to exhibit various adverse effects such as gastric pain, intestinal injuries and complication that involves the whole parts of the gastrointestinal tract [6–8]. Hence, search of novel anti-inflammatory agents with less or no toxic effect from natural sources is of interest.

Active metabolites such as proteins, phenolic compounds and antioxidants that were found in the soybean-based foods were reported to exhibit the anti-inflammatory effects [9]. One of the soybean-based foods that are largely consumed and incorporated in the daily diet in certain regions of Asia is fermented soybean. The fermentation process was able to generate an increased the level of soluble bioactive substances due to the microbial activities in soybean [10]. The benefits of antioxidants and amino acids, which are found to be substantially higher in fermented soybeans, are usually linked to the anti-inflammatory activity [11]. One of the most commonly consumed fermented soybeans around South East Asia is tempeh which contained significant level of antioxidants and amino acids [12, 13], which may subsequently contribute to the anti-inflammatory effect. Thus, the objectives of this study were to compare anti-inflammatory effect between Nutrient Enriched Soybean Tempeh (NESTE) aqueous extract and Soybean aqueous extract (SBE) through in vitro regulation of nitric oxide (NO) production in LPS-induced mice macrophage cells (RAW 264.7) and in vivo arachidonic acid induced mice ear edema as well as analgesic effect via in vivo hot plate test. Additionally, the acute toxicity of NESTE was also determined.

## Methods

### Chemicals and reagents

3-(4,5-dimethylthiazol-2-yl)-2,5-diphenyltetrazolium bromide (MTT), acetyl salicylic acid, dexamethasone, lipopolysaccharide (LPS), curcumin and Dulbecco’s Modified Eagle Medium (DMEM) were purchased from Sigma (USA), foetal bovine serum (FBS) from PAA (Austria), dimethyl sulfoxide (DMSO) from Fisher Scientific (UK) and arachidonic acid from Merck (USA).

### Animals

Five to six weeks old of male (*n* = 51) and female (*n* = 51) Balb/c mice with the average weight of 15 to 23 g were purchased from animal house, Faculty of Veterinary, Universiti Putra Malaysia. Mice were kept in plastic cage (3 or 4 mice per cage, autoclave sawdust) under controlled condition with a cycle of 12 h light and 12 h dark at 25 °C and were acclimatized for at least 1 week and supplied with standard diet pellet and water ad libitum. The experiments were approved by Institutional Animal Care and Use (IACUC) Committee, Universiti Putra Malaysia (UPM) (Ref: UPM/ FPV/ PS/ 3.2.1.551/AUP-R2) and compliance to the guidelines of the IACUC, UPM.

### Preparation of NESTE and SBE

Soybeans were obtained from the local market at Selangor, Malaysia, which was originated from Canada. Soybean samples was identified by the Science Officer Mr. Shaiful Sharifudin from MARDI and the voucher specimen (voucher no: MARBI 002) has been deposited at Biotechnology Research Center, MARDI. The starter culture of *Rhizopus* 5351 strain was obtained from the Bioprocess Department of Biotechnology Research Centre, Malaysian Agricultural Research and Development Institute (MARDI) and maintained at 4 °C for its growth. The preparation and extraction of raw soybean (SBE) and Nutrient-enriched soybean tempeh (NESTE) were carried out according to our previously published method [14]. Our previous study has reported that lyophilized NESTE contained 0.338 ± 0.025 g/100 g DW (dried weight) of gamma-aminobutyric acid (GABA), 2.176 ± 0.006 g/100 g DW of total free amino acids, 42.64 ± 1.59 μg/g extract of soluble phenolic acids and 22.56 ± 0.31 mg gallic acid equivalent (GAE)/g extract of total phenolic acids [14]. Total free amino acids and GABA were quantified using Waters Acquity Ultra Performance Liquid Chromatography (UPLC) by Acquity UPLC AccQ Taq Ultra Column and UV-photodiode array (PDA) detector set at 260 nm. On the other hand, soluble phenolic acids content was quantified by high-performance liquid chromatography (HPLC) using Chromolith Performance RP-18e column. In addition, total phenolic content was quantified by Folin-Ciocalteu reagent [14].

### In vitro anti-inflammatory activity

Mouse macrophage cell line (RAW264.7) was obtained from the American Type Culture Collection (ATCC) and maintained in Dulbecco’s Modified Eagle Medium (DMEM) supplemented with 2 mM glutamine, antibiotics (100 units/mL penicillin A and 100 units/mL streptomycin) and 10% heat-inactivated foetal bovine serum (FBS). The cells were then incubated in 5% CO_2_ at 37 °C.

The MTT assay was conducted according to Mosmann [15]. Briefly, 2 X 10^5^ cells/ mL of RAW264.7 cell was seeded in 96-well plate and allowed to attach overnight. Then, the cell was treated with various concentration of NESTE or SBE (0.63, 1.25, 2.50 and 5 mg/mL) for 24 h. After the incubation time, 20 μL of 5 mg/mL MTT was added to each well and 170 μL of media was aspirated after 3 h of incubation in the dark. Then, 100 μL of DMSO was added to each well to dissolve the crystal formazan and the optical density was measured at 570 nm by ELISA plate reader (Bio-Tek Instruments, USA).

The in vitro anti-inflammatory effect of SBE and NESTE were quantified by comparing the inhibition of extracellular secretion of nitric oxide (NO), IL-1β and TNF-α of LPS activated RAW264.7 cells after treated with 2.5 and 5 mg/mL of SBE or NESTE. Briefly, RAW264.7 cells were activated with 1 μg/ mL of LPS with or without the treatment of 2.5 and 5.0 mg/mL of SBE or NESTE. Curcumin (4 μg/mL) was used as positive anti-inflammatory compound control. After 24 h of incubation, of culture supernatant was collected for NO, IL-1β and TNF-α quantification. NO quantification was carried out using calorimetric Griess reaction kit (Molecular Probes, Inc., USA) according to Huang et al. [16], where 150 μL of supernatant was added with 20 μL of Griess Reagent and 130 μl of deionized water. Then, the reaction mixture was incubated for 30 min and the absorbance was measured at 548 nm wavelength using Bio-Tek ELISA (USA) plate reader. Standard calibration curve of sodium nitrite ranging from 1 to 100 μM was prepared concurrently and expressed as nitrite concentration (μM). The data was presented as percentage of nitric oxide inhibition. On the other hand, IL-1β and TNF-α were quantified using ELISA kit (BioLegend, USA) according to the manufacturer’s instructions. Each sample was assayed in triplicates.

### In vivo acute toxicity activity

Acute toxicity of SBE and NESTE was evaluated according to the OECD guideline [17]. Male and female ICR mice were divided into three groups where each group represents five males and five females in separate cages. The first group received distilled water (normal control), the second group received single oral dose 5000 mg/kg body weight of SBE while for the third group received single oral dose 5000 mg/kg body weight of NESTE (100 μL, dissolved in distilled water). After administration of the extracts (in the morning), mice were observed for abnormality and mortality for subsequent 1, 2, 4, 6, 24 h and up to days 14. At the end of the assessment, all the survived mice were anesthetized with 2% isoflurance (Merck, USA), sacrificed by cervical dislocation and the internal organs including liver, kidney and spleen were dissected, weight and fix for histopathology study using Hematoxylin and Eosin (H and E) staining [18]. In addition, the weight of the three organs (liver, kidney and spleen) was presented as organ index and measured as per below formula;

$$ \mathrm{Organ}\ \mathrm{index}=\mathrm{weight}\ \mathrm{of}\ \mathrm{mice}\ \mathrm{organ}\ \left(\mathrm{g}\right)/\mathrm{body}\ \mathrm{weight}\ \mathrm{of}\ \mathrm{mice}\ \left(\mathrm{g}\right)\ \mathrm{X}\ 100 $$

### Mice paw analgesia test

Hot plate method was used to induce pain for the mice paw according to Tapondjou et al. [19]. Hot plate was employed to introduce the heat to the mice and the response latencies were measured based on the time taken for the mice to start licking, shaking the hind paw or jumping. Briefly, mice were fasted for 24 h prior to experiment and randomly separated into 6 groups (*n* = 6, 3 male and 3 female). All mice were pre-treated orally (p.o.) with 100 μL of solution 30 min prior to placement on hot plate according to the following treatment. Group 1 represented mice fed with distilled water (negative control), group 2 represented mice treated with acetyl salicylic acid (400 mg/kg, dissolved in carboxymethylcellulose), group 3 and 4 represented mice treated with 200 mg/kg and 1000 mg/kg of SBE (dissolved in distilled water), respectively and group 5 and 6 represented mice treated with 200 mg/kg and 1000 mg/kg of NESTE (dissolved in distilled water), respectively. Thirty minutes after treatments, each mouse was put onto hot plate with a temperature of 55 °C individually and the response was monitored based on the mice reaction of paw licking or jumping. The latency until mice react by paw licking or jumping was recorded. This step was repeated at 60, 90 and 120 min after treatment to monitor the prolong analgesic effect of the treatment. Baseline (presented as 0 min) of each groups of mice was obtained by monitoring the reaction of mice on hot plate before the treatment. Data was expressed as the reduction number of response latency between control and extracts-treated mice.

### Statistical analysis

All the results were analysed using Statistical Package for the Social Sciences (SPSS version 18) (SPSS Inc., USA) software and expressed as means ± standard error mean (S.E.M). One-way analysis of variance (ANOVA) with Duncan post-hoc was used to obtain the mean differences and *p* value with less than 0.05 (*p* < 0.05) was considered as significant.

## Results

### Viability of RAW264.7 cells by MTT assay

**Fig. 1:**
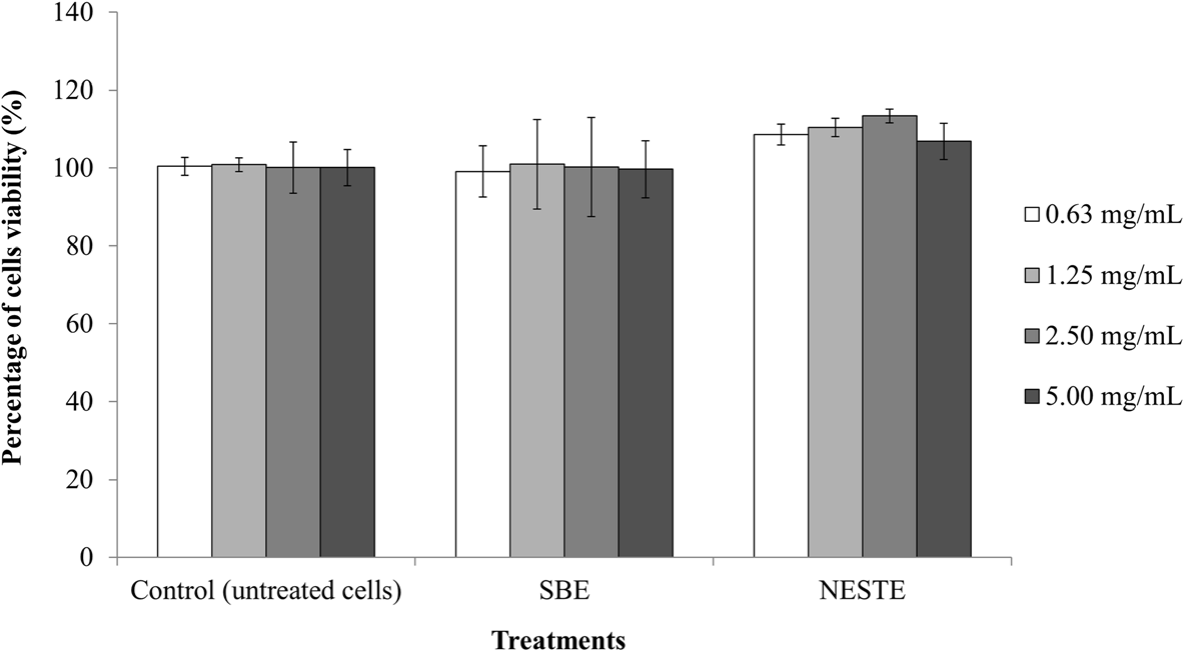
Percentage of RAW264.7 cells viability by MTT assay after treated with various concentration of NESTE and SBE for 24 h. Note: All of the values are expressed as means ± S.E.M. from three separate experiments

Figure 1 illustrated the effect of NESTE and SBE on viability of RAW 264.7 cell. Both NESTE and SBE did not induce cytotoxic effect toward RAW 264.7 cell even at the highest concentration of 5 mg/mL. Furthermore, both extracts have no significant differences with the control group (untreated cells). This result indicates that both NESTE and SBE did not influence the viability of RAW 264.7 cell.

### LPS-induced NO production in RAW 264.7 cells

**Fig. 2:**
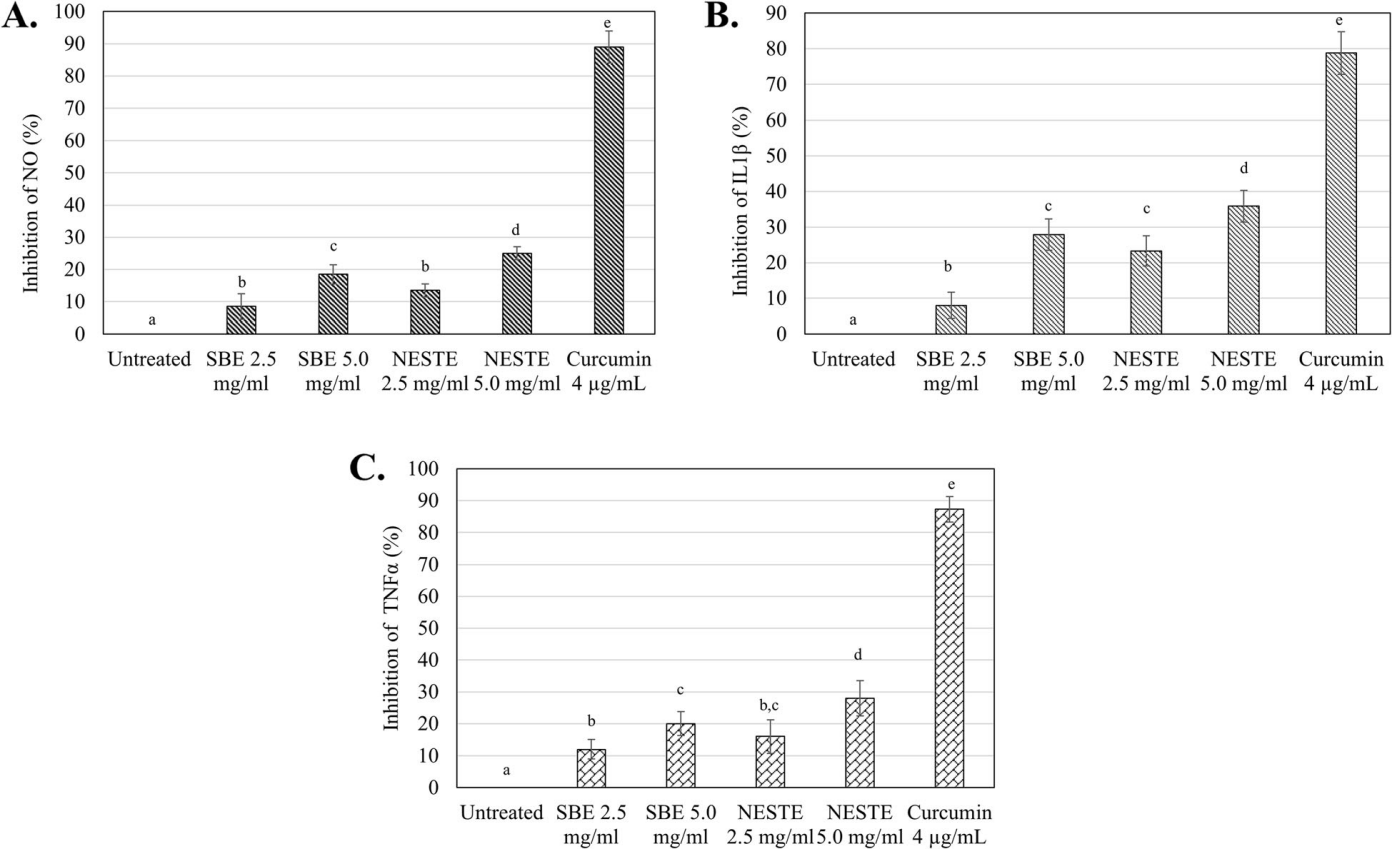
The effect of SBE and NESTE treatments towards the percentage of **a** nitric oxide; **b** IL1β; **c** TNFα inhibition on LPS-induced RAW264.7 cells. Note: All of the values are expressed as means ± S.E.M. from three separate experiments, ‘a’ value has a significance differences (*p* < 0.05) with ‘b’ value

The effect of NESTE and SBE on LPS-induced NO, IL-1β and TNF-α production in RAW 264.7 cell was summarized in Fig. 2. Both concentrations of NESTE and SBE inhibited secretion of the NO, IL-1β and TNF-α by the LPS activated RAW 264.7 after 24 h of incubation. Furthermore, 5 mg/mL of NESTE showed the highest inhibitory effect, ie 25.50 ± 2.20%, 35.88 ± 3.20% and 28.50 ± 3.50%, respectively on NO, IL-1β and TNF-α production by LPS treated RAW 264.7 cells as compared to 5 mg/mL of SBE (18.50 ± 2.0%, 27.80 ± 3.40% and 20.00 ± 2.30%, respectively), which indicated that fermentation process has significantly improved the in vitro anti-inflammatory effect of soybean. However, this effect is lower comparing to the inhibition of the reference compound 4 μg/mL of curcumin, which inhibited the NO, IL-1β and TNF-α production by 89.50 ± 5.00%, 78.80 ± 6.20% and 87.30 ± 4.00%, respectively.

### In vivo acute toxicity of NESTE and SBE

Single administration of 5000 mg/kg of NESTE and SBE was used in the acute toxicity study (OECD, 2001). All mice (*n* = 30) survived up to day-14 post-treated with SBE (*n* = 10) and NESTE (*n* = 10). Furthermore, no significant difference was observed in body weight, organ weight (spleen, liver and kidney) and organ histopathology (spleen, liver and kidney) (Data not shown) among the groups.

### In vivo analgesic effect of NESTE and SBE on mice by hot plate test

**Fig. 3:**
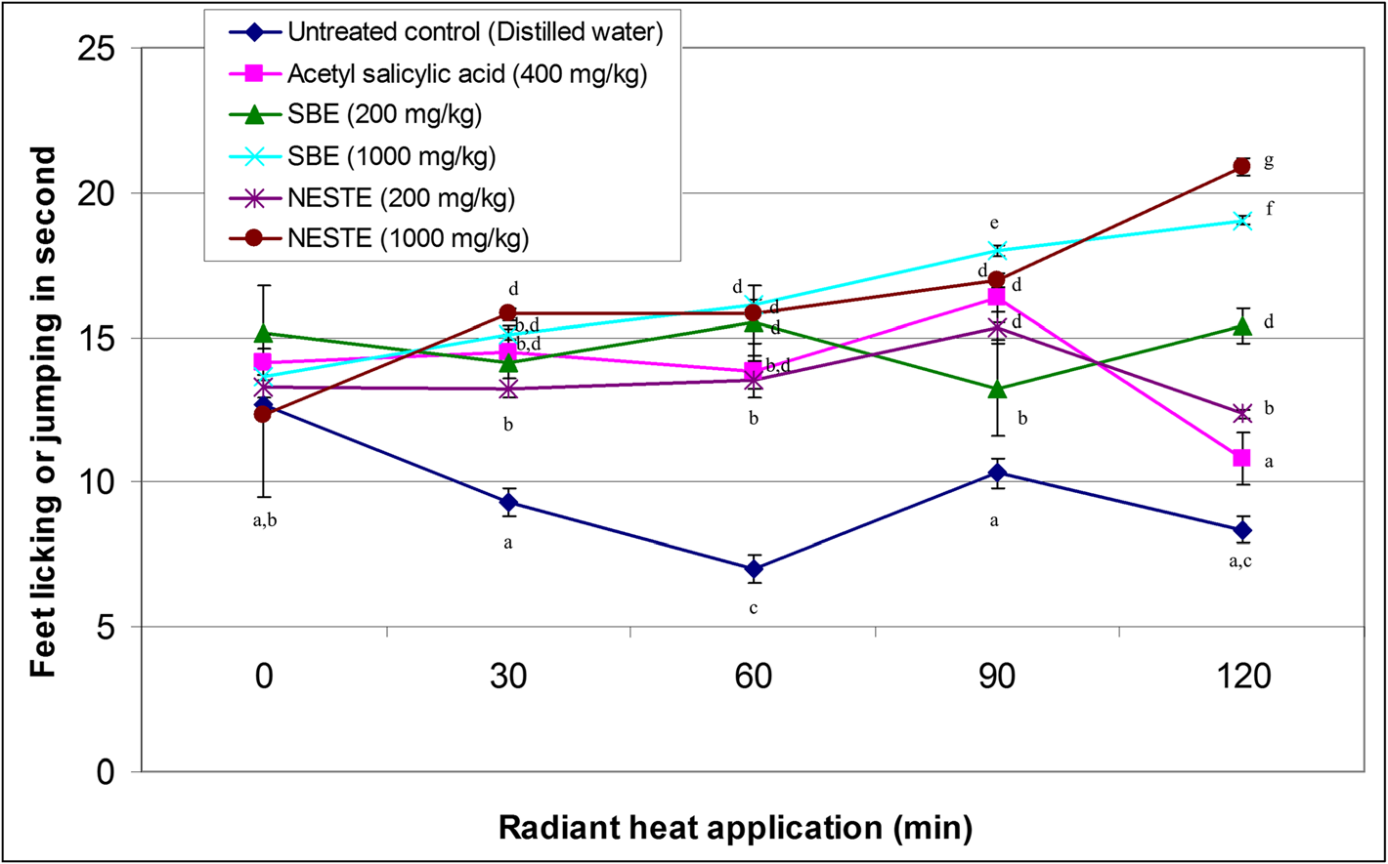
Analgesic activity of NESTE and SBE by hot plate method on mice. Note: The data represents as mean of licking/jumping for every 40 s ± S.E.M from six mice, Acetyl salicylic acid was used as reference substances

Analgesic effect of NESTE and SBE tested by the hot plate method in mice was summarized in Fig. 3. Mice treated with 400 mg/kg acetyl salicylic acid showed a significant analgesic effect showing by 1.7-fold increase of latency period than the untreated mice. However, this effect was only observed until 90 min. Nevertheless, 1.5 to 2.5-fold increase of latency period that last until 120 min of analgesic effect was shown in 1000 mg/kg NESTE treated mice where the effect demonstrated longer response for mice to resist the heat effect from hot plate up to 120 min.

## Discussion

Bacterial endotoxin lipopolysaccharides (LPS) activated mice macrophage cell lines (RAW 264.7) was commonly used as a model to investigate in vitro anti-inflammatory activity (Szliszka et al. 2011). Macrophage cells are generally known to have various pivotal roles in inflammatory diseases including discharging of inflammatory factors such as reactive oxygen species (ROS), reactive nitrogen species (RNS) and cytokines under the stimulation of immunostimulator such as bacterial endotoxin lipopolysaccharides (LPS) and interferon-gamma (IFN-γ) [3, 20]. The uncontrolled responses of these inflammatory factors could contribute to chronic inflammatory diseases including cellular dysfunction and tissues damage [5]. The significant bioactivity level of NESTE was proven to suppress the overexpression in the inflammatory activities. Furthermore, NESTE has no toxicity effect to the viability of RAW 264.7 cell line, which indicated that NESTE would not interferes the RAW 264.7 cell activity, as it simultaneously slows down the uncontrolled inflammatory responses. In the inflammatory reaction, macrophage generates nitric oxide (NO) which is usually detected as iNOS (inducible nitric oxide synthase) [21]. Generally, several functions in physiological and pathological activities have been reported in NO. However, chronic inflammatory diseases as well as autoimmune diseases could induce by an increase level of NO [4, 5]. NO and proinflammatory cytokines including IL-1β and TNF-α were involved in LPS induced inflammation of macrophages, which mimic the clinical sepsis-induced inflammatory responses. Suppression of NO production was reported with inhibition of LPS-induced IL-1β and TNF-α proinflammatory cytokines production [22]. The significant reduction of NO level was observed in the LPS induced RAW 264.7 cells treated with NESTE and this suggested that the effect of NESTE was comparable to the effect of fermented soymilk in the previous study, which had been revealed to inhibit the production of NO that subsequently suppressed the IL-1β and TNF-α proinflammatory cytokines production in LPS induced macrophage [11, 22]. Moreover, the hot plate test also demonstrated that NESTE treatment was the most efficient to induce the analgesic effect towards mice. These results suggested that NESTE potentially possess better in vitro anti-inflammatory and in vivo anti-analgesic effects than SBE. These results are similar with our previous report where NESTE was able to reduce NO level in the liver of mice treated with ethanol. This anti-inflammatory effect was correlated with the enhanced antioxidant level of the soybean through fermentation [23].

The prevalence of toxicity in fermented foods always correlate to the contaminants from microbial food-borne intoxication which was previously reported in majority of all types of fermented foods including cheese, sausages, fermented fish and fermented cereals [24]. Evaluation of the acute toxicity test in NESTE treatment group has shown that it has no acute toxicity and LD_50_ up to day 14. Furthermore, result of mice body weight gain, organs indices and histopathological examination in NESTE treatment group were shown to be comparable to the normal control group. These results were agreeable with the previous study, which revealed that tempeh is free from the hazard of food-borne intoxication [24].

Tempeh is a common fermented food in Asia including Indonesia, Malaysia, Thailand and Japan. Previous study has shown that daily recommended dosage was 250 g per person. In this study, the dose per 60 kg for human are 0.976 g/60 kg and 4.878 g/60 kg (based on the conversion of body surface area of mice to human) [25], respectively. As the extraction recovery for the tempeh extract was 25% [14], the amount of tempeh to achieve the tested dosage are 3.904 g/60 kg and 19.512/60 kg per person. This concentration is below the recommended daily dosage proposed by Nakajima et al. [26]. Better effect of NESTE maybe contributed by higher number of bioactive compounds detected in NESTE compared to SBE such as GABA by 328-fold, free amino acid by 32-fold, essential amino acid by 157-fold and TSPC by 2.3-fold. Furthermore, the overall flavonoids and antioxidant activities tested by total phenolic content, DPPH and FRAP assays exhibited significant increment [14]. Thus, we presumed that the augmented amount of these compounds may directly or indirectly contribute to the anti-inflammatory and analgesic effects. The findings were further supported by similar publications by other researcher on anti-inflammatory effect exhibited by such bioactive compounds, flavonoids [27], GABA [28], amino acids [29] and antioxidant activity [30]. Similarly, analgesic effects were shown in various other researches [31–33]. In addition, Mothana et al. [34] have claimed that antioxidant activity of plant flavonoids and phenolic acids is able to scavenge the reactive oxygen species (ROS), which are mainly involved in inflammation pathology [34].

## Conclusion

Increment of several bioactive compounds such as antioxidants, GABA, amino acids and phenolic acids in the NESTE extract produced from fermentation process using *Rhizopus* 5351 strain have significant anti-inflammatory and analgesic effects without causing acute toxicity in mice. Nevertheless, further studies are needed to assess the detailed mechanisms of individually isolated active ingredients in NESTE that contributed to these activities.

## Data Availability

The datasets used and/or analyzed during the current study available from the corresponding author on reasonable request.

## Abbreviations

DMEM: Dulbecco’s modified eagle medium
DMSO: Dimethyl sulfoxide
DPPH: 2,2-diphenyl-1-picrylhydrazyl
FBS: Foetal bovine serum
FRAP: Ferric reducing ability power
GABA: Gamma-aminobutyric acid
LPS: Lipopolysaccharide
MARDI: Malaysian Agricultural Research and Development Institute
MTT: 3-(4,5-dimethylthiazol-2-yl)-2,5-diphenyltetrazolium bromide
NESTE: Nutrient Enriched Soybean Tempeh
NO: Nitric oxide
NSAIDs: Non-steroidal anti-inflammatory drugs
RNS: Reactive nitrogen species
ROS: Reactive oxygen species
S.E.M.: Standard error mean
SBE: Soybean aqueous extract
UPM: Universiti Putra Malaysia
